# The Role of DPP-4 Inhibitors in the Treatment Algorithm of Type 2 Diabetes Mellitus: When to Select, What to Expect

**DOI:** 10.3390/ijerph16152720

**Published:** 2019-07-30

**Authors:** Konstantinos Makrilakis

**Affiliations:** National and Kapodistrian University of Athens Medical School, Laiko General Hospital, 17 Ag. Thoma St., 11527 Athens, Greece; kmakrila@med.uoa.gr; Tel.: +30-210-7462717 or +30-213-2061061

**Keywords:** Diabetes mellitus type 2, treatment, DPP-4 inhibitors

## Abstract

Type 2 diabetes mellitus is a growing global public health problem, the prevalence of which is projected to increase in the succeeding decades. It is potentially associated with many complications, affecting multiple organs and causing a huge burden to the society. Due to its multi-factorial pathophysiology, its treatment is varied and based upon a multitude of pharmacologic agents aiming to tackle the many aspects of the disease pathophysiology (increasing insulin availability [either through direct insulin administration or through agents that promote insulin secretion], improving sensitivity to insulin, delaying the delivery and absorption of carbohydrates from the gastrointestinal tract, or increasing urinary glucose excretion). DPP-4 (dipeptidyl peptidase-4) inhibitors (or “gliptins”) represent a class of oral anti-hyperglycemic agents that inhibit the enzyme DPP-4, thus augmenting the biological activity of the “incretin” hormones (glucagon-like peptide-1 [GLP-1] and glucose-dependent insulinotropic polypeptide [GIP]) and restoring many of the pathophysiological problems of diabetes. They have already been used over more than a decade in the treatment of the disease. The current manuscript will review the mechanism of action, therapeutic utility, and the role of DPP-4 inhibitors for the treatment of type 2 diabetes mellitus.

## 1. Introduction

Diabetes mellitus (DM) is a complex, chronic illness requiring continuous medical care with multifactorial risk-reduction strategies in order to reduce complications. The prevalence of the disease in adults is rapidly increasing worldwide. According to the International Diabetes Federation (IDF), in 2015 it was estimated that there were 415 million people aged 20–79 years living with diabetes on the planet, 5.0 million deaths were attributable to diabetes, and the total global health expenditure due to diabetes was estimated at 673 billion US dollars. The number of people with diabetes aged 20–79 years was predicted to rise to 642 million by the year 2040, the majority of who will be diagnosed with type 2 diabetes (T2D) [1]. The global costs of diabetes and its consequences are large and will substantially increase in the future [2]. Health care professionals and policy makers need to take urgent action to prepare health and social security systems to mitigate the effects of the disease. The good news lately is that significant evidence exists that supports a range of interventions to improve diabetes outcomes [3].

The pathogenesis of T2D is quite complex, mainly characterized by progressive beta cell dysfunction and insulin resistance [4]. Furthermore, other pathophysiological abnormalities [5], such as increased renal glucose re-absorption [6] and the diminished “incretin” effect [7] have been found to contribute to the hyperglycemia manifested by the disease. Due to the complexity of its pathophysiology, a number of different therapeutic options are available for its treatment. Apart from lifestyle modifications to reduce obesity through healthy diet and encouragement of physical activity, current pharmacologic therapeutic approaches are based upon increasing insulin availability (either through direct insulin administration or through agents that promote insulin secretion), improving sensitivity to insulin, delaying the delivery and absorption of carbohydrate from the gastrointestinal tract, or increasing urinary glucose excretion [8].

**Scheme 1:**
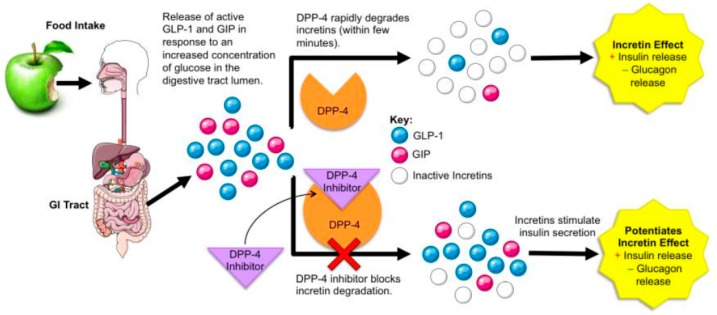
DPP-4 function and mechanism of DPP-4 inhibitors’ action.

Dipeptidyl-peptidase-4 (DPP-4) inhibitors (or ‘gliptins’) represent a class of oral anti-hyperglycemic agents that block the inactivation of the “incretin” hormones, namely glucagon-like peptide-1 (GLP-1) and glucose-dependent insulinotropic polypeptide (GIP), and thus affect glucose control through several mechanisms, including enhancement of glucose-dependent insulin secretion, slowed gastric emptying, and reduction of postprandial glucagon and of food intake (Scheme 1) [9]. Their role in the treatment of T2D will be reviewed in the current manuscript, with emphasis on their mechanism of action and therapeutic utility.

### 1.1. The “Incretin” Effect

The concept that oral nutrient (glucose) administration promotes a much greater degree of insulin secretion compared to a parenteral isoglycemic glucose infusion underlies the “incretin” (INtestinal seCRETion of INsulin) effect, namely the existence of gut-derived factors that enhance glucose-stimulated insulin secretion from the islet β-cell. The hypothetical existence of certain factors produced by the intestinal mucosa in response to nutrient ingestion that are capable of stimulating the release of insulin from the endocrine pancreas and thereby reducing blood glucose levels was first postulated in the early 1900s [10]. With the development of the radioimmunoassay, this communication between the intestine and the endocrine pancreas was confirmed when it was demonstrated that oral glucose administration is associated with a much greater increase in plasma insulin levels when compared to the same amount of glucose given intravenously [11]. The incretin effect is estimated to account for approximately 50%–70% of the total insulin secreted following oral glucose administration in normal persons [12].

The first incretin hormone to be identified was isolated from crude extracts of porcine small intestine and initially named gastric inhibitory polypeptide (GIP), based on its ability to inhibit gastric acid secretion in dogs. However, subsequent studies using more purified preparations of GIP revealed that it could also stimulate insulin secretion in animals and humans given in physiologic doses [13], and it was thus renamed “glucose-dependent insulinotropic polypeptide” to reflect its physiological action, yet retains the same acronym. GIP is a 42 amino acid peptide hormone that is released from K-cells of the small intestine, primarily in response to fat (and to a lesser extent glucose) ingestion, and potentiates glucose-stimulated insulin secretion [14] through a specific GIP receptor (GIPR) [15]. The majority of intestinal K cells are located in the proximal duodenum. Bioactive GIP has also been detected in pancreatic α-cells, raising the possibility of an intra-islet α-cell to β-cell connection.

The second incretin hormone that was identified nearly a decade later, glucagon-like peptide-1 (GLP-1), followed the cloning and sequencing of mammalian proglucagon genes and complementary DNAs. GLP-1 is a tissue-specific posttranslational proteolytic product of the proglucagon gene that is released from intestinal L-cells in response to nutrient ingestion and enhances glucose-stimulated insulin secretion in human subjects [16]. Glucagon-like peptide-1 is synthesized in intestinal endocrine cells in two principal major molecular forms, as GLP-1(7-36)amide and GLP-1(7-37), collectively referred to as intact bioactive GLP-1. The majority of circulating biologically active GLP-1 is found in the GLP-1(7-36)amide form, with lesser amounts of the bioactive GLP-1(7-37) form also detectable [17,18]. GLP-1 is constantly secreted from the intestine at low basal levels in the fasted and interprandial state, and circulating GLP-1 levels increase ≈ 2- to 3-fold after nutrient ingestion. It plays multiple roles in metabolic homeostasis following nutrient absorption. The biological activities of GLP-1 include stimulation of glucose-dependent insulin secretion and insulin biosynthesis, inhibition of glucagon secretion and gastric emptying, and inhibition of food intake. GLP-1 appears to have a number of additional effects in the GI tract and central nervous system as well, leading to delayed gastric emptying [19] and promoting satiety [20].

These two peptides (GLP-1 and GIP) potentiate glucose-stimulated insulin secretion in an additive manner, likely contribute equally to the incretin effect and together can fully account for the majority of the incretin effect in humans. Diabetes is associated with one or more defects in the incretin axis, but it is not yet clear whether these defects contribute to the development of type 2 diabetes or arise as a consequence of hyperglycemia and or other metabolic manifestations of diabetes itself. Early studies demonstrated that the incretin effect was diminished in subjects with type 2 diabetes [21]. This reduced incretin effect has been attributed to defective GIP action and reduced GLP-1 secretion. Newer studies however, do not support the contention of a generalized defect in nutrient-related GLP-1 secretory responses in people with T2D, but rather suggest that the inter-individual differences in GLP-1 secretion, age of patients, duration of diabetes, concomitant drug therapy, and other variables play a role, and preclude definitive conclusions that diabetes is invariably associated with reduced GLP-1 secretion [22]. The majority of studies indicate that defective GLP-1 secretion does not appear to predate the development of glucose intolerance, rather diabetes itself seems to be associated with the acquired defect in GLP-1 secretion and GIP action [23].

Comparison of the actions of infused GLP-1 and GIP in human subjects with T2D, irrespective of the etiology of the disease, demonstrates that although the initial early phase of insulin secretion in response to GIP is preserved in diabetic subjects, the later phase from 20–120 min is characterized by a completely defective insulin response to GIP, even when higher concentrations of GIP are infused in study subjects, whereas the insulin secretory response to GLP-1 is preserved [24]. The finding that GLP-1 lowers blood glucose in patients with diabetes, taken together with suggestions that GLP-1 may restore β-cell sensitivity to exogenous secretagogues, suggests that augmenting GLP-1 signaling is a useful strategy for treatment of diabetic patients [25].

GLP-1 binds to a specific GLP-1 receptor (GLP-1R), which is expressed in various tissues, including pancreatic beta cells, pancreatic ducts, gastric mucosa, kidney, lung, heart, skin, immune cells, and the hypothalamus [26]. GLP-1 exerts its main effect by stimulating glucose-dependent insulin release from the pancreatic islets [27]. Although the primary biological actions ascribed to intact GLP-1 are mediated by a single GLP-1 receptor [28], GLP-1 is rapidly cleaved by the enzyme dipeptidyl peptidase-4 (DPP-4) into GLP-1(9-36) [29], which exhibits its own biological activities (albeit not insulin secretion any more) [30,31], and thus intact GLP-1 exhibits a short half-life of one to two minutes in the circulation [32]. DPP-4 inhibitors prevent or attenuate the normal degradation of GLP-1(7-36) amide to GLP-1(9-36) amide. Several studies have raised the possibility that GLP-1(9-36) amide, which is proportionately more abundant than the full length bioactive peptide, may not simply represent an inert cleavage product, but may function either as an endogenous GLP-1R antagonist or a weak agonist [33,34], or as a unique agonist with insulin-independent glucose-lowering properties [35]. The importance of GLP-1(9-36)amide has been assessed in healthy humans, where elegant studies have shown no effect of this peptide on insulin secretion or glucose clearance [36]. GIP is also cleaved by DPP-4 into inactive GIP(3-42) [30].

### 1.2. The DPP-4 Enzyme

Dipeptidyl peptidase-4 (DPP-4), originally described as a lymphocyte cell surface protein (identical to the T-cell activation antigen cluster of differentiation (CD)-26), is a ubiquitously expressed transmembrane-spanning glycoprotein exopeptidase of 110 kDa, that exerts biological activity through pleiotropic actions. The enzyme is also known to be cleaved-off from the cell membrane (involving different metalloproteases in a cell-type specific manner, by a process called “shedding”) and thus to also exist in a circulating, soluble form (sDPP-4) [37]. Both membrane-associated and soluble DPP-4 exert catalytic activity, cleaving proteins containing a position 2 alanine or proline. Despite the preference for a position 2 proline, alternate residues (hydroxyproline, dehydroproline>alanine> glycine, threonine, valine, or leucine) at the penultimate position are also cleaved by DPP-4, suggesting a required stereochemistry [38]. The DPP-4 cleavage at postproline peptide bonds inactivates peptides and/or generates new bioactive peptides, thereby regulating diverse biological processes. The complexity of DPP-4 action is amplified by the panoply of bioactive DPP-4 substrates, including cytokines, growth factors, neuropeptides and the incretin hormones [39], which in turn act as elegant biochemical messengers in multiple tissues, including the immune and neuroendocrine systems [40]. DPP-4 is widely expressed in numerous tissues including endothelial cells in multiple vascular beds, rendering the enzyme highly accessible to peptide substrates circulating through the gut, liver, lung, and kidney [41].

DPP-4 substrates are classified as pharmacological or physiological [37]. A physiological DPP-4 substrate is defined as one whose relative levels of intact versus cleaved peptide are significantly different in an animal or human treated with a DPP-4 inhibitor, or in an animal with genetic inactivation of DPP-4 (e.g., GIP, GLP-1, GLP-2, Peptide tyrosine tyrosine [PYY], Stromal cell-derived factor-1 [SDF], and Substance P [SP]). In contrast, a pharmacological substrate is cleaved by DPP-4 ex vivo, but evidence for cleavage of the endogenous peptide in vivo may be lacking (e.g., aprotinin, BNP, β-casomorphin, chorionic gonadotrophin, endomorphin-1, endomorphin-2, enterostatin, eotaxin, erythropoietin, GCSF [granulocyte colony stimulating factor], and many others). Given the low circulating and tissue levels of most DPP-4 substrates and the technical challenge inherent in quantifying intact versus cleaved peptides, it seems likely that some DPP-4 substrates currently classified as pharmacological may one day also be found to be cleaved by DPP-4 in vivo [20].

Since DPP-4 rapidly cleaves and inactivates the incretin hormones (GLP-1 and GIP), which are essential for glucose regulation, its blockade has been investigated as a way of ameliorating glycemia in diabetes through preservation of the impaired incretin action [7]. DPP-4 is a member of the serine peptidase/prolyl oligo-peptidase gene family, which includes the membrane-bound peptidases, fibroblast activation protein (FAP)/seprase; the resident cytoplasmic enzymes, DPP8 and DPP9; and the nonenzymatic members, DPP6 and DPP10, which are present in neuronal membranes, and prolyl endopeptidase [37]. Biological interest in the DPP-4 enzyme has heightened after the discovery and approval of highly selective DPP-4 inhibitors (agents that selectively inactivate DPP-4 and augment the incretin effect) for the treatment of type 2 diabetes [42,43].

### 1.3. DPP-4 Inhibitors

There are five DPP-4 inhibitors in the market today, including sitagliptin, saxagliptin, linagliptin, alogliptin (in the United States and Europe), and vildagliptin (only available in Europe) [44]. Four more gliptins, namely teneligliptin, anagliptin, omarigliptin, and trelagliptin are only approved in the Japanese and Korean markets.

Despite the same mode of action, the various gliptins have differences in their pharmacodynamic and pharmacokinetic properties, which might be clinically relevant for some patients (Table 1) [45]. Their efficacy though, both in terms of inhibiting plasma DPP-4 activity and as antidiabetic agents, appears to be similar [44]. The main differences between them include: potency, target selectivity, oral bioavailability, elimination half-life, binding to plasma proteins, metabolic pathways, formation of active metabolite(s), main excretion routes, dosage adjustment for renal and liver insufficiency, and potential drug-drug interactions [46].

Sitagliptin was the first agent to be approved for the treatment of type 2 diabetes by the FDA on 17 October 2006 (for use as monotherapy, or combination therapy, with either metformin or a thiazolidinedione). Saxagliptin was the second one to be approved in 2009 and the others followed thereafter.

DPP-4 inhibitors are orally administered once per day, with the exception of vildagliptin which is dosed twice per day. They demonstrate a significant inhibition of plasma DPP-4 activity within 5 min of administration. Given the lack of a clinically significant increase with a high fat meal, they can be taken without regard to food [47]. Oral biovailability in humans is generally high (∼87% for sitagliptin [48], 85% for vildagliptin [49] and ∼67% for saxagliptin [50], although somewhat lower for linagliptin (∼30%) [44,51].

Regarding pharmacokinetic, pharmacodynamic properties and excretion of the compounds [52], most of the inhibitors display low, reversible protein binding in the plasma, (38% for sitagliptin [48], 10% for vildagliptin [53] and negligible for saxagliptin [44]). In contrast, linagliptin binds extensively to plasma proteins in a concentration-dependent manner and it has been calculated that at the therapeutic dose (5 mg) most of the drug will be protein bound (primarily to DPP-4) [54]. Generally, the DPP-4 inhibitors are eliminated primarily via the kidney, with the rate of renal clearance exceeding glomerular filtration, suggesting that active transport is involved [53,55,56]]. Linagliptin is the exception, with <6% of the dose being excreted in the urine [51]. This may be, at least in part, because of the high degree of protein binding [54], meaning that the drug escapes glomerular filtration. Rather, linagliptin has a hepatic route of elimination, with 78% appearing in the feces unchanged. Renal excretion of the primary metabolite of linagliptin (CD1790) is negligible; this undergoes further effects of the metabolism and is also eliminated in the feces [57].

Since DPP-4 is a member of a family of proteases (FAP, DPP-8/DPP-9, DPP-6, DPP-10, etc) [37], in order to minimize any potential off-target side effects, the inhibitors intended to be used therapeutically have to be selective for this specific enzyme. Thus, in this respect, sitagliptin and alogliptin can both be described as being highly selective; they essentially show no inhibitory activity against other members of the DPP-4 family when tested in vitro [58,59]. Vildagliptin and saxagliptin are somewhat less selective with regard to inhibition of DPP-8/9 in vitro [60], although whether this has any significance in vivo is questionable because DPP-8/9 are located intracellularly. Linagliptin, while being selective with regard to DPP-8/9, is less selective with regard to fibroblast activation protein-α (FAPα)/seprase. FAPα is an extracellular enzyme which is not generally present in normal adult tissue (although it is expressed in stromal fibroblasts and up-regulated during tissue remodeling). However, the extent of any FAPα inhibition in vivo with the therapeutic dose of linagliptin in humans has not been reported [44].

With a few exceptions, there are no significant drug–drug interactions when gliptins are co-administered with a variety of commonly prescribed medications, such as statins [61]. Because cytochrome P450 isoenzyme CYP3A4/5 metabolizes saxagliptin to its primary metabolite, strong CYP3A4/5 inhibitors, such as diltiazem, ketoconazole, and ritonavir, may increase saxagliptin exposure and thus one should consider dose reduction when co-administering these compounds. P-glycoprotein and CYP3A4 inducers, such as rifampicin, may decrease the efficacy of linagliptin. Furthermore, a reduction in the dose of sulfonylureas is usually recommended when a DPP-4 inhibitor is added, because of a pharmacodynamic interaction (rather than a pharmacokinetic interaction) between the sulfonylurea and the DPP-4 inhibitor, which may result in a higher risk of hypoglycemia.

There is a paucity of direct head-to-head studies between the different DPP-4 inhibitors regarding their clinical efficacy and/or adverse effects. Most comparisons are indirect, in different studies, and in different populations [62].

### 1.4. Glycemic Efficacy

The principle of using DPP-4 inhibitors as therapy of T2D is now firmly established [43]. Inhibition of DPP-4 prevents the degradation of GLP-1 and GIP and enhances glucose-stimulated insulin secretion (incretin action). GLP-1 and GIP act on the pancreatic β-cell to increase insulin release. GLP-1 also acts on the α-cell to suppress glucagon release and ultimately suppress hepatic glucose production. Together, the increased cellular glucose uptake and the decreased hepatic glucose output offer physiologic glucose control with the use of DPP-4 inhibitors and justifies their use in treating T2D.

DPP-4 inhibitors are not recommended for use as initial monotherapy for T2D treatment [8,63]. They are most commonly prescribed in combination with lifestyle modification and metformin, sulfonylureas, thiazolidinediones, and/or basal insulin, but selected patients intolerant to or with contraindications for metformin (such as patients with chronic kidney disease) can be successfully treated with DPP-4 inhibitor monotherapy. However, their modest glycemia-lowering effectiveness and relative expense temper enthusiasm for these drugs. There are a number of combination products available, including gliptin-metformin, gliptin-pioglitazone and gliptin-sodium glucose co-transporter-2 inhibitor products (Table 1).

The DPP-4 inhibitors appear to have similar glycemic efficacy [64]. They result in modest improvement in glycated hemoglobin (HbA1c), with a reduction of ~0.5–1% when used as monotherapy and ~0.6%–1.1% when used in combination with metformin, depending on agent, dose of therapy, and starting HbA1c. However, there are few head-to-head trials and no clinical trial data on long-term (greater than 3 years) safety, mortality effect, diabetic complications, or health-related quality of life. In an 18-week trial of saxagliptin (5 mg/day) versus sitagliptin (100 mg/day) in 800 patients inadequately controlled on a stable dose of metformin, there were similar reductions in HbA1c (−0.52 vs. −0.62 percentage points) [65]. In addition, the results from a meta-analysis of studies comparing sitagliptin with placebo or vildagliptin with placebo suggest similar efficacy (weighted mean difference in HbA1c values of −0.74 and −0.73%, 95% CI −0.84 to −0.63 and −0.94 to −0.52, for sitagliptin and vildagliptin compared with placebo, respectively) [66]. In a study comparing vildagliptin to sitagliptin using 24-h continuous glucose monitoring, both demonstrated similar reductions in HbA1c, fasting plasma glucose and post-prandial glucose, but the magnitude of glycemic changes (based on mean amplitude of glycemic excursions [MAGE]) decreased more significantly with vildagliptin therapy [67]. In a subsequent similar study, there was no difference regarding MAGE between the two drugs, but vildagliptin induced better circadian glycemic control than sitagliptin with a significant decrease on overall hyperglycemia, mainly driven by reduction on basal hyperglycemia [68]. The clinical significance of these findings is unknown.

DPP-4 inhibitors have been evaluated as monotherapy and in various combinations with other glucose-lowering agents, and compared with either a placebo or an agent of another glucose-lowering pharmacological class as an active comparator [69]. They generally exert slightly less pronounced HbA1c reduction than metformin (with the advantage of better gastrointestinal tolerability) and similar glucose-lowering effects as with a thiazolidinedione (TZD; with the advantage of no weight gain). In metformin-treated patients, gliptins were associated with similar HbA1c reductions compared with a sulfonylurea (SU; with the advantage of no weight gain, considerably fewer hypoglycemic episodes and no need for titration) and a TZD (with the advantage of no weight gain and better overall tolerability). In fact, although a recent meta-analysis combining the results of long-term clinical trials suggested that the effect of DPP-4 inhibitors on HbA1c levels in patients with T2DM may decline during the second year of treatment [70], direct head-to-head comparison of glycemic durability of DPP-4 inhibitors and sulfonylureas showed that DPP-4 inhibitors were associated with significantly smaller increases in the HbA1c level from 24 to 28 weeks to 104 weeks (mean difference [MD]: −0.16%, 95% confidence interval [CI]: −0.21 to −0.11; *p* < 0.001) and from 52 weeks to 104 weeks (MD −0.06%, 95% CI −0.10 to −0.02; *p* = 0.001), suggesting that longer-term treatment (over 2 years) with DPP-4 inhibitors confers slightly better durability of glycemic response than treatment with SUs [71]. DPP-4 inhibitors also exert clinically relevant glucose-lowering effects compared with a placebo in patients treated with SU or TZD (of potential interest when metformin is either not tolerated or contraindicated), and as oral triple therapy with a good tolerability profile when added to a metformin-SU or pioglitazone-SU combination. Several clinical trials also showed a consistent reduction in HbA1c when DPP-4 inhibitors were added to basal insulin therapy, with no increased risk of hypoglycemia [62,72]. However, DPP-4 inhibitors are less effective than GLP-1 receptor agonists for reducing HbA1c and body weight [73], but offer the advantage of being easier to use (oral instead of injected administration) and being lower in cost [74]. Clearly, more trials of direct comparisons between different incretin-based therapies are needed.

### 1.5. Cardiovascular Effects

All agents for treatment of hyperglycemia in diabetes are evaluated for efficacy in lowering blood glucose levels and overall safety and are subjected to particularly rigorous screening for cardiovascular safety through randomized controlled trials. The approach to these cardiovascular studies is largely directed by a Guidance for Industry issued by the FDA in 2008 stating that all new type 2 diabetes drug development programs should rule out unacceptable cardiovascular risk by demonstrating an upper bound of the two-sided 95% CI of the risk ratio <1.8 pre-approval for a composite end point of major adverse cardiac events (MACE), consisting of at least cardiovascular death, nonfatal myocardial infarction, and stroke. For drugs in which the upper bound of the CI is between 1.3 and 1.8, the guidance states that subsequent post-approval trials of the same composite MACE end point should be conducted to continue marketing [75].

Up to now, the FDA-mandated randomized controlled cardiovascular outcome trials (CVOTs) regarding all four DPP-4 inhibitors currently in clinical use in the United States have been completed and published. They have shown that DPP-4 inhibitors have generally been safe regarding atherosclerotic cardiovascular events (or the development or progression of kidney disease) in T2D patients.

Since vildagliptin is not available in U.S.A., no randomized control CVOT study has been performed, but a manufacturer-sponsored meta-analysis of a large pool of studies, including trials in high-risk patients with T2D, such as those with congestive HF and/or moderate/severe renal impairment showed that vildagliptin was not associated with an increased risk of adjudicated MACEs relative to comparators or a significantly increased risk of HF in vildagliptin-treated patients [76]. This was confirmed in subsequent independent meta-analyses [77]. Furthermore, the VIVID (Vildagliptin in Ventricular Dysfunction Diabetes) study reported that diabetic patients with heart failure receiving vildagliptin showed no adverse effect on ejection fraction compared to placebo. Though the primary endpoint indicated that vildagliptin did not have an unfavorable effect on left ventricular ejection fraction, there was in fact an increase in left ventricular end-diastolic volume (*p* = 0.007) and a 14% decrease in BNP in the vildagliptin group, suggesting that the increased left ventricular (LV) volumes observed did not result in increased LV wall stress [78].

In the saxaglitpin SAVOR-TIMI 53 trial, 16,492 diabetic subjects with a history of myocardial infarction (MI), or documented atherosclerosis, or at least one of hypertension, smoking, or dyslipidemia and HbA1c 6.5–12% were randomized to receive saxagliptin or placebo, in addition to other diabetes medications (predominantly metformin, sulfonylurea and insulin) [79]. Additional anti-diabetic agents were prescribed at the discretion of the treating physician throughout the study. The primary endpoint was a composite of cardiovascular death, non-fatal MI or non-fatal ischemic stroke. Median follow-up time was 2.1 years and maximum follow up 2.9 years. No differences in cardiovascular endpoints were observed in saxagliptin vs. placebo-treated patients (HR 1.00, 95% CI 0.89–1.12). No differences in multiple safety endpoints, including pancreatitis or cancer, were observed across groups. Unexpectedly, hospitalization for heart failure was more common in saxagliptin-treated subjects (3.5% vs. 2.8%; HR 1.27; 95% CI 1.07–1.51; *p* = 0.007). The significance of the increased risk for hospitalization for heart failure with saxagliptin is poorly understood. Subjects at greatest risk of hospitalization for heart failure had previous heart failure, an estimated glomerular filtration rate ≤60 mL/min, or elevated baseline levels of N-terminal pro B-type natriuretic peptide (NT-pro BNP). There was no evidence of heterogeneity between NT-pro BNP and saxagliptin (*p* for interaction = 0.46), although the absolute risk excess for heart failure with saxagliptin was greatest in the highest NT-pro-BNP quartile (2.1%) [80].

In the EXAMINE (Examination of cardiovascular outcomes with alogliptin) study, 5380 patients with a recent (15–90 days) MI or unstable angina requiring hospitalization were randomized to alogliptin or placebo and followed for a median of 18 months (up to 40 months). No differences in the primary endpoint (composite of death from cardiovascular causes, non-fatal MI or non-fatal stroke) was observed between groups (HR 0.96, upper boundary of the one-sided CI 1.16). Rates of pancreatitis, cancer, and hypoglycemia, were also similar between groups [81]. Given the heart failure results of the SAVOR-TIMI 53 trial with saxagliptin, in a post hoc analysis of the EXAMINE data, more patients in the alogliptin group were hospitalized for heart failure (3.9% vs. 3.3% in the placebo group), albeit this was a non significant increase (HR 1.19, 95% CI 0.90–1.58). Alogliptin had no effect on composite events of cardiovascular death and hospital admission for heart failure in the post hoc analysis (HR 1.00, 95% CI 0.82–1.21) and results did not differ by baseline BNP concentration [82].

In the sitagliptin TECOS study, the cardiovascular safety of sitagliptin vs. usual diabetes care was examined in 14,671 patients with T2D and established CVD (history of major coronary artery disease, ischemic cerebrovascular disease, or atherosclerotic peripheral arterial disease) over ~3 years. Sitagliptin-treated patients experienced a slightly greater reduction in HbA1c (0.3%). The primary composite cardiovascular outcome (cardiovascular death, nonfatal myocardial infarction, nonfatal stroke, or hospitalization for unstable angina) occurred in a similar proportion of patients (11.4 and 11.6% in the sitagliptin and placebo groups, respectively; HR 0.98, 95% CI 0.89–1.08). There was no significant difference in any of the individual components of the composite endpoint. There was no significant difference in the rate of hospitalization for heart failure either (3.1% in each group) [83].

In the linagliptin CARMELINA trial, 6991 patients with type 2 diabetes and at high CVD and renal risk were randomly assigned to linagliptin or placebo, in addition to other diabetes medications (predominantly metformin, sulfonylurea and insulin) [84]. After a median follow-up of 2.2 years, the primary outcome (first occurrence of the composite of cardiovascular death, nonfatal myocardial infarction or nonfatal stroke) occurred in a similar proportion of patients (12.4 vs. 12.1% for placebo, HR 1.02, 95% CI 0.89–1.17). There was no difference in the secondary kidney outcome (composite of end-stage renal disease, death due to renal failure, or a sustained decrease of at least 40% in eGFR from baseline), which occurred in 9.4% and 8.8% of patients in the linagliptin and placebo groups, respectively (HR 1.04, 95% CI 0.89–1.22). There was no significant difference in the rate of hospitalization for heart failure (6.0% vs. 6.5% with placebo) [85].

Findings from the Cardiovascular Outcome Study of Linagliptin Versus Glimepiride in Patients With Type 2 Diabetes (CAROLINA), recently presented (10 June 2019) at the American Diabetes Association (ADA) 2019 Scientific Sessions, showed that there were no major differences in cardiovascular outcomes between the DPP-4 inhibitor linagliptin and the sulfonylurea glimepiride among patients with early type 2 diabetes at increased cardiovascular risk. CAROLINA is the first cardiovascular outcomes trial to include an active comparator and it provided valuable information about the safety of both linagliptin and glimepiride in treating T2D patients.

The clinical significance of the finding of increased hospitalization for heart failure, mainly in the saxagliptin, and less so in the alogliptin study, is unclear [32,86,87,88]. The relative effect of DPP-4 inhibitors on the risk of heart failure in patients with T2D is uncertain, given the relatively short follow-up and low quality of evidence. Both randomized controlled trials and observational studies, however, suggest that these drugs may increase the risk of hospital admission for heart failure in those patients with existing cardiovascular diseases or multiple risk factors for vascular diseases, compared with no use [89]. The FDA has issued a warning since 2016 and recommends discontinuation, initially specifically of saxagliptin and alogliptin, and later also for sitagliptin, in patients who develop heart failure, and monitoring to determine if alternative therapy for diabetes is required.

### 1.6. Mortality

DPP-4 inhibitors do not appear to have any effect on overall mortality. A systematic meta-analysis of 189 trials showed no difference in all-cause mortality between any incretin drug versus control [90]. In a subgroup analysis of the DPP-4 cardiovascular outcomes trials, there was no difference in all-cause mortality between a DPP-4 inhibitor and placebo (6.1 versus 6.0%, OR 1.02, 95% CI 0.91–1.14).

### 1.7. Adverse Effects

The DPP-4 inhibitors as a class appear to be very well tolerated, and rates of adverse effects have been low, and generally not different from a placebo or comparator. However, although the DPP-4 inhibitors are relatively specific for GLP-1, the long-term consequences of DPP-4 inhibition and its effects on other DPP-4 substrates are unknown. Due to the ubiquitous nature of dipeptidyl peptidase substrates and the variable specificity of DPP-4 inhibitors, each agent within this class will need to be scrutinized individually for drug-specific side effects, especially in the long-run [91]. It is possible that the risk of side effects may be higher with less selective DPP-4 inhibitors. Residual crossover with other substrates of DPP-4, particularly with respect to immune function, remains a concern, although this has not been reported in short-term clinical trials and the CVOTs so far.

The DPP-4 inhibitors have neutral effects on body weight or risk of hypoglycemia (justified by the glucose-dependent insulin secretion mode of action of GLP-1) [62,92], except when there is concomitant treatment with insulin or sulfonylureas [93].

An early meta-analysis of incretin based therapies (in which inhibitor data were available only for sitagliptin and vildagliptin), suggested that there was an increased risk of some infections (urinary tract infections with both inhibitors and nasopharyngitis more evident with sitagliptin) and headache (more evident with vildagliptin) [66,94]. Since then, updated safety analyses (each > 10,000 patients, exposed for up to 2 years) of the sitagliptin and vildagliptin clinical trials have been published, showing no increased risk for urinary tract or respiratory infections or headache (and indeed, no increased risk of any other adverse effect) with the DPP-4 inhibitors compared to placebo or comparator [95,96]. A subsequent meta-analysis of trials (18 to 104 weeks’ duration) comparing a DPP-4 inhibitor (sitagliptin, saxagliptin, vildagliptin, linagliptin, alogliptin) with placebo (44 trials), a comparator from another class of antidiabetic agents (20 trials), or another DPP-4 inhibitor (three trials) showed a small increased risk of nasopharyngitis compared with placebo (6% vs. 5.3%, RR 1.13, 95% CI 0.99–1.29), which was predominantly driven by the sitagliptin subgroup (5.3% vs. 4.1%; RR 1.35, 95% CI 1.03–1.77) [97]. The risk of upper respiratory and urinary tract infections was not significantly elevated, whereas the risk of dizziness and headache was slightly elevated (8.2% vs. 7.5%; RR 1.14, 95% CI 1.02–1.26).

At the time of initial registration of vildagliptin (in EU), a meta-analysis of the clinical trial data revealed that the 100 mg qd dose was associated with small numerical elevations in liver transaminases compared to placebo or 50 mg bid. For this reason, the recommended therapeutic dose was changed to 50 mg bid, with the recommendation that liver function tests be performed before initiation and at three monthly intervals for the first year of treatment and periodically thereafter. Subsequently, the trend for mild increases (greater than three times the upper limit of normal) in liver enzymes was confirmed in the larger pooled safety analysis, but notably, this was not associated with any increased incidence of actual hepatic adverse events [97]. Nevertheless, liver function tests are still recommended and vildagliptin is not approved for use in patients with hepatic insufficiency.

Acute pancreatitis has been reported in association with DPP-4 inhibitors [98], but a causal relationship has not been established [99,100]. In large cohort studies, current use of incretin-based therapy was not associated with an increased risk of hospitalization for acute pancreatitis [101] or pancreatic cancer [102]. Since in some studies though, an arithmetically higher incidence of acute (but not chronic) pancreatitis has been found [103], pancreatitis should be considered in patients with persistent severe abdominal pain (with or without nausea), and DPP-4 inhibitors should be discontinued in such patients. If pancreatitis is confirmed, a DPP-4 inhibitor should not be restarted. In addition, DPP-4 inhibitors should not be initiated in a patient with a history of pancreatitis. After a review of currently available data, the US Food and Drug Administration (FDA) and the European Medicines Agency agreed that there was insufficient evidence to confirm an increased risk of pancreatitis and pancreatic cancer with use of GLP-1-based therapies [104]. However, based also on small histopathologic studies that pointed to a marked expansion of the exocrine and endocrine pancreatic compartments with the use of incretin-based therapies, the former being accompanied by increased proliferation and dysplasia and the latter by α-cell hyperplasia with the potential for evolution into neuroendocrine tumors [105], concerns remain [106], and monitoring for and reporting of pancreatic adverse effects will continue [104].

Use of DPP-4 inhibitors has been associated with an increased risk of inflammatory bowel disease compared with other diabetes drugs (53.4 vs. 34.5 per 100,000 person-years, HR 1.75, 95% CI 1.22–2.49) in a population-based study [107]. These findings require confirmation and further investigation into possible mechanisms.

During postmarketing surveillance, serious allergic reactions, including anaphylactoid reactions, angioedema [108], and exfoliate dermatologic reactions (such as Stevens-Johnson syndrome), were reported. These reactions have typically occurred within 3 months of sitagliptin initiation, with some occurring after the first dose. Among clinical trial recipients who received 2.5 or 5 mg saxagliptin daily, alone or in combination with metformin, a thiazolidinedione, or glyburide, 1.5% had a hypersensitivity-related event such as urticaria and facial edema (angioedema) compared with 0.4% in the placebo recipients [109]. Vildagliptin use has also been associated with increased risk of angioedema among patients taking ACE inhibitors, although the absolute risk is small [110]. Use of DPP-4 inhibitors has been associated with an at least doubling of the risk of bullous pemphigoid in patients with type 2 diabetes, albeit the absolute risk is again very low [111].

Some DPP-4 inhibitors (sitagliptin, vildagliptin, saxagliptin) have been associated with severe joint pain [112]. Other reported musculoskeletal side effects include myalgias, muscle weakness, and muscle spasms. Symptoms have been reported from two days to five months after initiating DPP-4 inhibitors. In most patients, symptoms resolved within a month after discontinuing the drug. Some patients developed recurrent severe joint pain after restarting the same or a different DPP-4 inhibitor. The FDA released a safety announcement in 2015 which alerted providers to report severe and persistent joint pain with use of DPP-4 inhibitors. Sitagliptin was the most frequently reported, followed by saxagliptin > linagliptin/vildagliptin > alogliptin. The FDA recommendation is to stop the DPP-4 inhibitor if there is any suspicion of causation, and that reintroducing the same agent or another in the class was associated with recurrence of symptoms in some patients [113].

### 1.8. Use in Special Populations

#### Patients with Renal Insufficiency

Because most of DPP-4 inhibitors are eliminated renally, it is expected that their pharmacokinetic profile will be influenced by impairments in renal function and that their plasma levels will increase in proportion to renal failure [44]. Indeed, exposure to sitagliptin increased proportionately to the degree of renal failure [114]. Despite that, the drug was well tolerated, even in patients with end-stage renal disease (ESRD), including those on dialysis. Based on this study, it was concluded that no dose adjustment was necessary in subjects with mild renal insufficiency [creatinine clearance (CrCl) 50–80 mL/min]. However, in order to maintain plasma sitagliptin exposure comparable to that in subjects with normal renal function, in subjects with moderate renal insufficiency (CrCl 30–50 mL/min) or severe renal insufficiency (CrCl < 30 mL/min)/ESRD, dose reductions of 50 and 75%, respectively, are required. In T2D patients with moderate or severe chronic renal insufficiency (including those with ESRD on dialysis), sitagliptin provided effective glycemic control over 1 year and was generally well tolerated [115].

Exposure to vildagliptin and saxagliptin is also similarly increased in subjects with impaired renal function [53,116], and like sitagliptin, vildagliptin is reported to be well tolerated in patients with mild renal insufficiency, with the rate of adverse events being similar to comparators [97] (Table 2). On the basis of these observations, sitagliptin, vildagliptin and saxagliptin have been approved for use in subjects with mild renal insufficiency without dose adjustment, and where the indication is approved, they can be used in patients with moderate or severe renal insufficiency/ESRD with appropriate dose adjustment (Table 2). Alogliptin is also eliminated renally and can be used in subjects with moderate or severe renal impairment with dose reduction [117,118] (Table 2).

Because linagliptin is not predominantly eliminated via the kidneys, it can be anticipated that this drug can be used in renal disease patients, including those with severe renal insufficiency/ESRD without the need for any dose adjustment [119,120].

Serum creatinine should be measured prior to initiating DPP-4 inhibitors, and then every three to six months in patients with eGFR ≤45 mL/min/1.73m^2^ and approximately every 6 to 12 months in those with eGFR >45 mL/min/1.73m^2^.

### 1.9. Elderly Persons with T2D

Several studies have shown a high prevalence of T2D in the elderly [121]. Frequently, T2D in the elderly is diagnosed when a complication occurs, among which are cognitive disorders and/or affective disturbances. Moreover, hypoglycemia is a frequent side effect of therapeutic treatment with insulin, sulfonylureas or glinides, while other treatments (metformin, acarbose, thiazolidinediones, GLP-1 receptor agonists, and DPP-4 inhibitors) are capable of reducing hyperglycemia without inducing hypoglycemia. Thus, considering that older persons are a very heterogeneous group of individuals, management of T2D in the elderly is challenging. Because of their pharmacokinetic characteristics, pharmacodynamic properties (glucose-dependent glucose-lowering effect) and good overall tolerability profile, DPP-4 inhibitors may have a key role to play in elderly patients [122].

Other than the surrogate endpoint of improved glycemic control though, data on clinically relevant benefits of DPP-4 inhibitors in the treatment of type 2 diabetes mellitus in older adults is scarce. DPP-4 inhibitors might have a lower risk of hypoglycemia compared to other antidiabetic drugs but data show conflicting findings for long-term benefits. Further studies are needed to guide specific recommendations [123].

## 2. Conclusions

Dipeptidyl peptidase-4 (DPP-4) inhibitors are oral diabetes medications that inhibit the enzyme DPP-4, a ubiquitous enzyme that is expressed on the surface of most cell types and deactivates a variety of other bioactive peptides, including glucose-dependent insulinotropic polypeptide (GIP) and glucagon-like peptide-1 (GLP-1). DPP-4 inhibitors thus affect glucose regulation by augmenting the incretin system. Given the large number of substrates cleaved by DPP-4 with diverse effects particularly in the immune and endocrine systems, the long term safety of DPP-4 inhibitors in human studies merits careful consideration and analysis. Furthermore, each one of the DPP-4 inhibitors is a unique chemical entity and may exhibit a profile of adverse events specific to that chemical entity, which may not be generalizable as a “class effect”.

Generally, the DPP-4 inhibitors comprise of a group of chemically diverse compounds, which differ in terms of their potency to inhibit the DPP-4 enzyme, their duration of action and their metabolism and elimination, as well as isolated compound-specific characteristics [44]. They are all apparently well tolerated (side-effect profile resembles placebo) and result in clinically meaningful reductions in blood glucose (fasting and postprandial) and HbA1c levels, with minimal risk of hypoglycemia and without weight gain. They are used without the need for dose titration and give broadly similar HbA1c lowering efficacy to other oral antidiabetic agents. At present, although there are some practical differences between the different DPP-4 inhibitors with respect to dosing frequency and their ability to be used in different patient subpopulations, there seems to be little to distinguish between them in terms of their efficacy as antidiabetic agents and their safety. In particular, linagliptin might be a good choice as initial therapy in a patient with chronic kidney disease at risk for hypoglycemia. Other DPP-4 inhibitors may be used in the setting of chronic kidney disease with proper dose adjustment.

On the other hand, their long-term benefits and durability of glycemic effects remain unclear. Hence, they are not recommended as initial therapy for treatment of T2D. They do appear to be a reasonable second-line add-on therapy to metformin, especially in individuals at high risk for hypoglycemia (i.e. elderly) or in whom a weight sparing or oral regimen is preferred. However, the degree of HbA1c lowering desired must be taken into account, and the relatively modest glycemic effects at lower starting HbA1c, as well as their cost compared to other agents may temper one’s enthusiasm for prescribing this class of therapy. Additionally, in high cardiovascular risk patients, the cardiac effects have been neutral, and other classes of antidiabetic medications (in particular SGLT-2 inhibitors or GLP-1 agonists) are preferred [63]. The risk of hospitalization for heart failure remains poorly understood, and risk factors such as prior heart failure and chronic kidney disease should be considered when prescribing this class of therapy [47].

## Figures and Tables

**Table 1:** Comparison of currently marketed DPP-4 inhibitors.

Drug	Dosage Forms	Dosage Change in Renal Dysfunction	Dosage Change in Hepatic Dysfunction	Excretion	DPP-4 Inhibition	Half Life (Hours)	Metabolism	Available in Fixed-Dose Combination
Sitagliptin	25 mg	Yes	No	Renal (∼80% unchanged as parent)	Max ∼97%; >80% 24 h post-dose	8–24	Not appreciably metabolized	With metformin, With simvastatin
	50 mg							
	100 mg							
Saxagliptin	2.5 mg	Yes	No	Renal (12–29% as parent, 21–52% as metabolite)	Max ∼80%; ∼70% 24 h post-dose	2–4 (parent) 3–7 (metabolite)	Hepatically metabolized to active metabolite (via P450 3A4/5)	With metformin, With dapagliflozin
	5 mg							
Vildagliptin	50 mg	Yes	Not recommended in severe dysfunction. Liver testing before administration	Renal (22% as parent, 55% as primary metabolite)	Max ∼95%; >80% 12 h post-dose	1½–4½	Hydrolysed to inactive metabolite (P450 enzyme independent)	With metformin
	Dose:							
	50mg bid;							
	50 mg qD in eGFR <45 ml/min							
Alogliptin	6.25 mg	Yes	No	Renal (>70% unchanged as parent)	Max ∼90%; ∼75% 24 h post-dose	12–21	Not appreciably metabolized	With metformin, With pioglitazone
	12.5 mg							
	25 mg							
Linagliptin	5 mg	No	No	Biliary (>70% unchanged as parent); <6% via kidney	Max ∼80%; ∼70% 24 h post-dose	10-40	Not appreciably metabolized	With metformin,
								With empagliflozin

**Table 2:** Dosage of DPP-4 inhibitors in renal and hepatic insufficiency.

Drug	Renal Insufficiency			Hepatic Insufficiency	
	Mild (CrCl ≥ 50 mL/min)	Moderate (CrCl ≥ 30–<50 mL/min)	Severe/ESRD (CrCl < 30 mL/min)	Mild/Moderate	Severe
Sitagliptin	100 mg/d	50 mg/d	25 mg/d	100 mg/d	Not recommended #
Saxagliptin	5 mg/d	2.5 mg/d	Not recommended	5 mg/d	Not recommended
Vildagliptin *	50 mg bid	50 mg/d	50 mg/d	Not recommended	Not recommended
Alogliptin	25 mg/d	12.5 mg/d	6.25 mg/d	25 mg/d	Not recommended #
Linagliptin	5 mg/d	5 mg/d	5 mg/d	5 mg/d	Not recommended #

CrCl: creatinine clearance; ESRD: end-stage renal disease; * Assessment of hepatic function recommended prior to initiation of vildagliptin and periodically thereafter; # No clinical experience.

## Abbreviations List:

DPP-4	dipeptidyl-peptidase-4;
GLP-1	glucagon-like peptide-1;
GIP	glucose-dependent insulinotropic polypeptide;
DM	diabetes mellitus;
IDF	International Diabetes Federation;
T2D	type 2 diabetes;
BNP	Brain natriuretic peptide;
GCSF	granulocyte colony stimulating factor;
FAP	fibroblast activation protein;
CYP	cytochrome P450 isoenzyme;
FDA	Food and Drug Administration;
MACE	major adverse cardiac events;
CVOTs	cardiovascular outcome trials;
LV	left ventricle;
MI	myocardial infarction;
NT-pro BNP	N-terminal pro B-type natriuretic peptide;
EU	European Union;
CrCl	creatinine clearance; ESRD: end-stage renal disease.
